# Triangulated Cylinder Origami-Based Piezoelectric/Triboelectric Hybrid Generator to Harvest Coupled Axial and Rotational Motion

**DOI:** 10.34133/2021/7248579

**Published:** 2021-02-19

**Authors:** Jihoon Chung, Myunghwan Song, Seh-Hoon Chung, Woojin Choi, Sanghyun Lee, Zong-Hong Lin, Jinkee Hong, Sangmin Lee

**Affiliations:** ^1^School of Mechanical Engineering, Chung-Ang University, 84, Heukseok-ro, Dongjak-gu, Seoul, Republic of Korea; ^2^Department of Chemical & Biomolecular Engineering, College of Engineering, Yonsei University, 50 Yonsei-ro, Seodaemun-gu, Seoul 03722, Republic of Korea; ^3^Institute of Biomedical Engineering and Department of Power Mechanical Engineering, National Tsing Hua University, 101, Section 2, Kuang-Fu Road, Hsinchu 30013, Taiwan

## Abstract

Piezoelectric nanogenerators (PENGs) and triboelectric nanogenerators (TENGs) are representative technologies that can harvest mechanical energy. In general, piezoelectric/triboelectric hybrid generators can harvest considerable energy with a limited input; however, PENGs and TENGs entail different requirements for harvesting energy. Specifically, PENGs produce a large output when a large mechanical strain is applied, and TENGs require a large surface area to produce a high power. Therefore, it is necessary to develop an innovative strategy in terms of the structural design to satisfy the requirements of both PENGs and TENGs. In this study, we developed a triangulated cylinder origami-based piezoelectric/triboelectric hybrid generator (TCO-HG) with an origami structure to enable effective energy harvesting. The proposed structure consists of a vertical contact-separation TENG on the surface of the triangulated cylinder, PENG on the inner hinge, and rotational TENG on the top substrate to harvest mechanical energy from each motion. Each generator could produce a separate electrical output with a single input. The TCO-HG could charge a 22 *μ*F commercial capacitor and power 60 LEDs when operated.

## 1. Introduction

Energy harvesting devices have been extensively researched over the past decade, and the number of low-power electronic devices has increased considerably. These devices convert ambient power sources, such as sunlight [1–4], heat [5–8], and RF [9, 10], into useful electricity; further, they can be utilized as independent power sources or self-powered sensors that emit electric signals. Among varied ambient power sources, mechanical energy is an abundant energy source that can be harvested through Maxwell's displacement current (polarization-induced current) [11]. Notably, piezoelectric nanogenerators (PENGs) and triboelectric nanogenerators (TENGs), which utilize the polarization-induced current to produce electricity, are representative technologies for harvesting mechanical energy [12–15]. These two generators exhibit distinctive advantages such as a low weight, high electric output, and possibility of customizing the device design [16–20]. To produce a larger amount of electrical energy from a limited mechanical input, piezoelectric/triboelectric hybrid generators, which exhibit higher energy conversion efficiency, have been developed [19, 21–23]. However, the requirements for obtaining the maximum outputs of PENGs and TENGs are different. The electrical output of PENGs is based on the mechanical deformation of piezoelectric materials; therefore, PENGs produce a high output when a large mechanical strain, such as compression or bending, is applied [24, 25]. In contrast, the energy harvesting mechanism of TENGs is primarily dependent on the surface charge of the material, and thus, a larger surface area is necessary to produce a higher power [26–28]. Therefore, to realize effective hybrid mechanical energy harvesting, an innovative strategy must be developed in terms of the structural design to satisfy the requirements of both PENGs and TENGs.

Among various structural design methods, origami, which is a Japanese concept for folding paper, can be a potential strategy to design three-dimensional (3D) structures [29–31]. In particular, by folding a surface into a 3D shape, the surface area in a limited 3D space can be maximized, and the mechanical deformation of the substrate can be increased by folding [32]. In addition, by controlling the geometry of the origami substrate, the substrate can be arranged to move in specific directions [33, 34]. Moreover, by combining a mechanical hybrid generator with an origami structure, on-demand design can be achieved for each generator.

In this study, we developed a triangulated cylinder origami-based piezoelectric/triboelectric hybrid generator (TCO-HG) consisting of a vertical-type TENG, rotational-type TENG, and PENG. The triangulated cylinder (TC) was composed of identical triangles that formed a 3D cylindrical shape. Through this TC structure, the requirements of a high mechanical deformation for the PENG and large surface area for the TENG could be satisfied. When the TCO-HG is vertically compressed, it collapses in the vertical direction and rotates around the vertical axis simultaneously. In the case of a five-sided TC, the surface contact area of a single floor of the TC was 1.38 times larger than that of a flat surface, and the TC had five hinges that could be folded. On each triangular surface of the TC, a vertical contact TENG was implemented to maximize the surface contact area, and the PENG was fabricated on the hinges to induce the mechanical deformation of the piezoelectric material. Moreover, TCO-HG could be stacked to a multifloored TC to further increase the surface contact area and rotation angle. When vertical pressure was applied to the device, the vertical contact TENG, PENG, and rotational TENG could separately produce electrical power.

## 2. Result and Discussion

**Figure 1:**
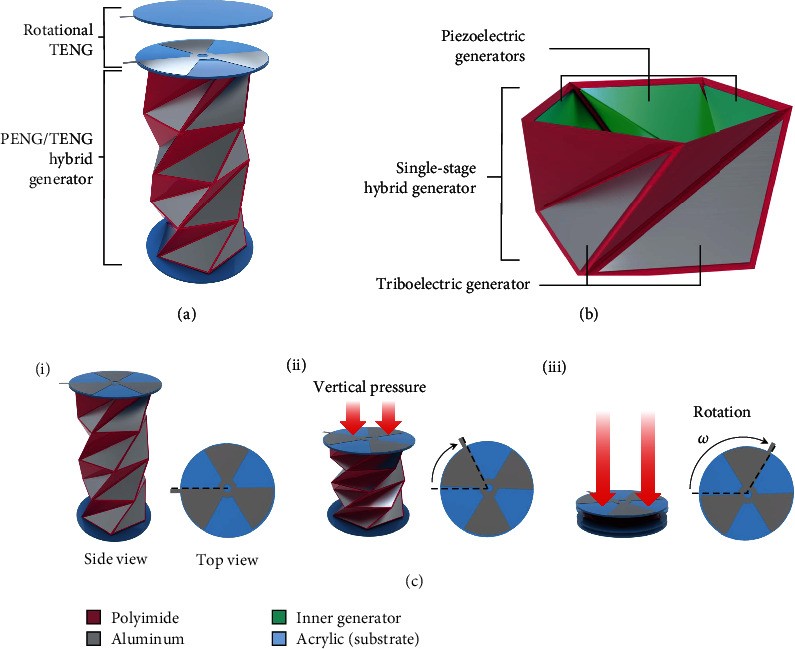
Schematic of triangulated cylinder (TC) origami-based piezoelectric/triboelectric hybrid generator (TCO-HG). (a) TCO-HG composed of rotational triboelectric nanogenerator (TENG) and piezoelectric nanogenerator (PENG)/TENG hybrid generator. (b) Single-stage hybrid generator composed of TENG and PENG on the outer and inner surfaces, respectively. (c) Rotation of top substrate during application of external pressure.

Figure 1(a) shows the schematic of the TCO-HG, which consists of a rotational TENG and PENG. The rotational TENG has three electrodes and is shaped as a 52° sector. When the TCO-HG is compressed, the top substrate rotates, and the rotational TENG on the top surface produces electrical energy. In the TCO-HG, four stages of the hybrid generator were connected to realize rotational motion when the device was compressed. The schematic of a single-stage hybrid generator is shown in Figure 1(b). TENGs and PENGs were fabricated on each outer and inner side of the TC surface, respectively. As the base of the TC was a regular polygon, except for the equilateral triangle shape [35], the number of TENGs and PENGs varied depending on the number of sides of the TC base. Owing to its high elasticity and surface charge, polyimide was selected as the substrate for the device. The substrate rotated during the compression of the TC (Figure 1(c)). As shown in Figure 1(c), the four-stage TCO-HG could rotate up to 104.97° when completely compressed [36]. To harvest the rotational energy from compression, a rotational TENG was fabricated on the top substrate [37].

The working mechanism of vertical contact-separation TENG in the hybrid generator is schematically illustrated in Figure S1. The vertical contact-separation TENG is made as single-electrode TENG which has single electrode on the bottom triangle surface of TC [38, 39]. As the top triangular surface approaches to the bottom electrode, the electrons flow to the electrical ground due to the negatively charged surface of polyimide. As the top polyimide surface detaches from the bottom electrode, the electrons flow back to the electrode creating alternative current as the top surface continues to move back and forth.

**Figure 2:**
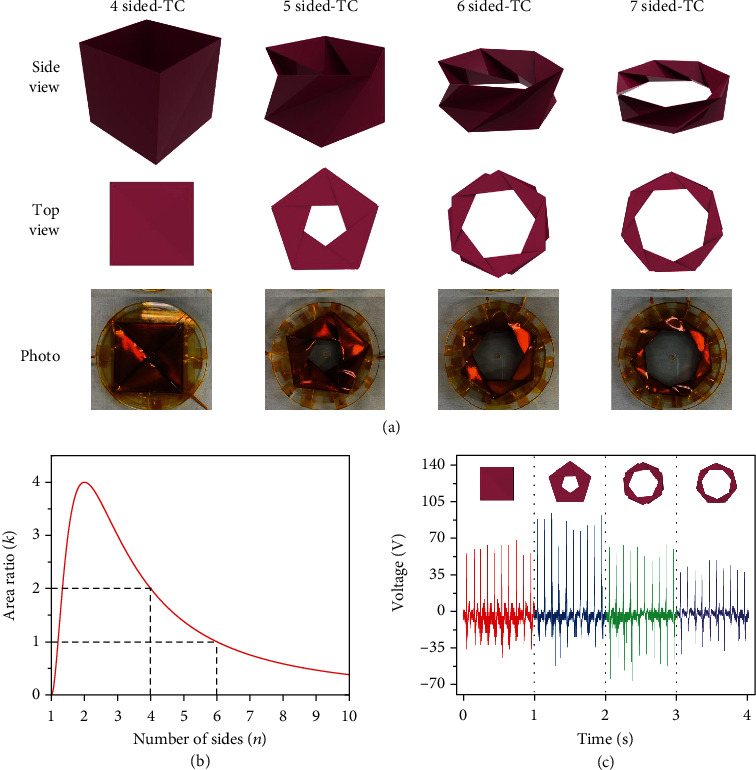
Vertical TENG in the TCO-HG. (a) Schematics and photographs of four-, five-, six-, and seven-sided TCs. (b) Relationship between number of sides (*n*) and area ratio (*k*). (c) Open-circuit voltage of four-, five-, six-, and seven-sided TCs.

By folding the surfaces through certain methods, the surface area of an origami structure can be maximized in a limited space. The TC has a regular polygon base except for the equilateral triangle shape. As shown in the top view in Figure 2(a), different TCs were fabricated with different base shapes, such as a square (four-sided TC), pentagon (five-sided TC), hexagon (six-sided TC), and heptagon (seven-sided TC). As shown in the schematic and image of the TC, the TC substrate involved an isosceles triangle forming a polygon shape, with a polygon-shaped empty space in the middle. Because the electrical performance of the TENG is proportional to the contact area of the device, the area ratio (*k*) between the contact area and regular polygon must be identified to maximize the contact area.

In general, *k* can be obtained geometrically. For a regular polygon composed of isosceles triangles, shown in Figure S2a, the area of a single isosceles triangle can be defined as

(1) $$\begin{array}{c} A_{1}=\frac{1}{2}\alpha l, \end{array}$$

where *A*_1_ is the area of the isosceles triangle shown in Figure S2a, *α* is the length of one side of the isosceles triangle, and *l* is the height of the isosceles triangle. Depending on the number of sides of the regular polygon (*n*), the area of a regular polygon (*A*_reg_) can be defined as

(2) $$\begin{array}{c} A_{\text{reg}}=\frac{n}{2}\alpha l. \end{array}$$

By definition, if the interior angle of a polygon is expressed as *θ*_1_ = (*n* − 2)/*πn*, *θ*_2_ can be expressed as 2*π*/*n*, and *l* can be expressed as

(3) $$\begin{array}{c} l=\frac{\alpha }{2}\cot\left(\frac{\theta_{2}}{2}\right)=\frac{\alpha }{2}\cot\left(\frac{\pi }{n}\right). \end{array}$$

By combining equations (2) and (3), *A*_reg_ can be expressed as

(4) $$\begin{array}{c} A_{\text{reg}}=\frac{n}{4}\cot\left(\frac{\pi }{n}\right)\alpha^{2}. \end{array}$$

As mentioned previously, the TC structure consists of an isosceles triangle forming a polygon as shown in Figure S2b. The minimum *n* for a TC is 4 (*n* ≥ 4). The interior angle of the polygon (*θ*_1_) can be expressed as (*n* − 2)/*πn*, and the area of a single isosceles triangle can be defined as

(5) $$\begin{array}{c} A=\frac{1}{2}\alpha^{2}\sin\theta_{1}. \end{array}$$

Moreover, the area of a one-floor TC with *n* isosceles triangles can be defined as

(6) $$\begin{array}{c} A_{\text{ori}}=\frac{n}{2}\alpha^{2}\sin\theta_{1}=\frac{n}{2}\sin\left(\frac{n-2}{n}\pi \right)\alpha^{2}=\frac{n}{2}\sin\left(\frac{2\pi }{n}\right)\alpha^{2}. \end{array}$$

Therefore, by combining equations (4) and (6), *k* can be expressed as

(7) $$\begin{array}{c} k=\frac{A_{\text{ori}}}{A_{\text{reg}}}=\frac{2\sin\left(2\pi /n\right)}{\cot\left(\pi /n\right)}=\frac{4\sin\left(\pi /n\right)\cos\left(\pi /n\right)}{\cot\left(\pi /n\right)}=4\sin^{2}\left(\frac{\pi }{n}\right)=2-2\cos\left(\frac{2\pi }{n}\right). \end{array}$$

Figure 2(b) shows the relationship between *k* and *n*, according to equation (7). As shown in the plot, *k* is 2 and 1 when *n* is 4 and 6, respectively. In other words, the surface area of a six- and seven-sided TC is the same and 0.75 times smaller than that of a flat polygon, respectively.

Figure 2(c) shows the open-circuit voltage output performance of *n*-sided TCs, with *n* ranging from 4 to 7. It can be observed that the voltage output of the five-sided TC is higher than that of the other TCs. Although the area ratio of a four-sided TC is higher than that of the other TCs, as shown in Figure 2(a), the four-sided TC is completely filled and does not have any empty space. To fabricate the TC substrate for the TCO-HG, a certain offset for the substrate thickness must be implemented for a smooth operation. However, as the four-sided TC does not have any space for the material, the substrate is deformed after only one compression and does not restore to its original height. The peak frequency is the same between each *n*-sided TC because each side of TC contacts all at once when input pressure is applied. Figure S3 shows an image of the deformed four-sided TC after one cycle of pressure application. After this stage, the device moves only slightly and generates a smaller electrical output compared to that for a five-sided TC.

In addition, owing to the elasticity of the substrate material, the TC does not fold completely, especially near the hinge, as shown in Figure S4a. To ensure the foldability of the TCO-HG, we fabricated a slit on the hinge (Figure S4b-i). As shown in Figures S4b-ii and S4b-iii, the foldability of TCO-HG is significantly enhanced compared to that of the nonslit sample, as shown in Figures S4a-ii and S4a-iii.

**Figure 3:**
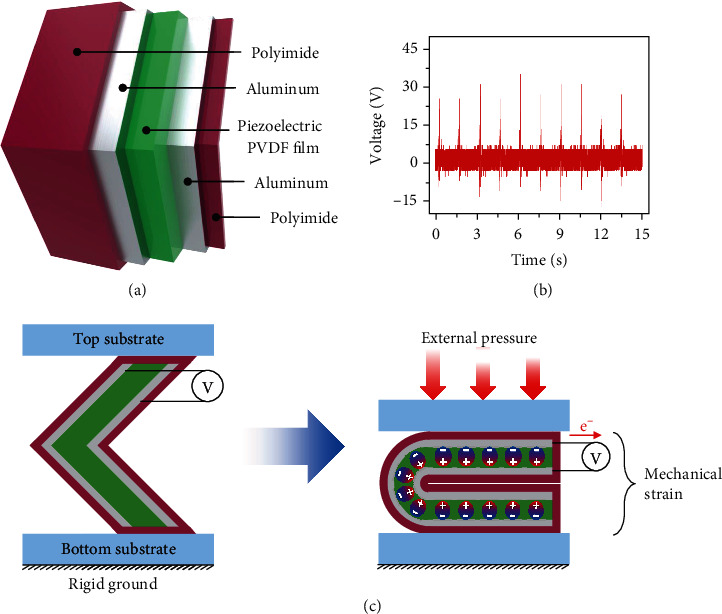
Inner PENG in the TCO-HG. (a) Structural schematic of inner PENG. (b) Open-circuit voltage of the PENG during operation. (c) Electrical potential difference generated under the application of external pressure.

On the back side of the TC substrate, a PENG is fabricated using a piezoelectric poly(vinylidene) difluoride (PVDF) film (Figure 3(a)). The PENG has an aluminum-piezoelectric PVDF film-aluminum sandwich structure on a polyimide substrate, covered with a thin polyimide film for protection. Polymorphous PVDF exhibited different electrical properties depending on the polymer chain conformations, i.e., *α*-phase, *β*-phase, and *γ*-phase (Figure S5a). In detail, the fraction of electroactive *β*- and *γ*-phase (*F*_EA_) positively correlated with the piezoelectric property of synthesized PVDF [40]. In this study, we maximized *F*_EA_ controlling the processing parameters, solvent properties of good solvent and nonsolvent. Based on the Hansen solubility parameters (*δ*), DMSO and water were, respectively, determined as the good solvent, nonsolvent of PVDF (Table S1) [41]. Since RED≈0.0 is the metric for the good solvent of specific polymer, DMSO was adapted to dissolve PVDF. Due to the high dipole moment (ca. 4.1), DMSO could result in sufficient electroactive phase content compared with other good solvents, such as DMF, MEK, and THF [42].

On the other hand, the coagulation bath was full of water (RED > 1) to induce the phase separation of as-dissolved PVDF. Further, two strategic modifications were applied to induce more electroactive phases, i.e., low precipitation temperature and high ionic concentration. The significant thermal gradient inside PVDF could be formed by quenching at -20°C. Thus, *F*_EA_ could be increased due to the sequential crystallization from nonsolvent/PVDF interface [43]. Based on the hydrogen bonds and dipole-dipole interactions, positively charged ions facilitated the phase inversion from *α*-phase to *β*- or *γ*-phase [44].

We prepared two PVDF films using a general water bath at room temperature and an ionic coagulation bath at low temperature. In Figure S5b, *F*_EA_ of PVDF films was measured using FT-IR and the following equation.

(8) $$\begin{array}{c} F_{\text{EA}}=\frac{A_{\text{EA}}}{\left(K_{\text{EA}}/K_{\text{nEA}}\right)A_{\text{nEA}}+A_{\text{EA}}},\left(\text{S}1\right) \end{array}$$

where EA is the electroactive phase (*β*- and *γ*-phase), nEA is the nonelectroactive phase (*α*-phase), and *K* is the absorption coefficient at each phase (in detail, *K*_EA_ ≈ 7.7 × 10^4^; *K*_nEA_ ≈ 6.1 × 10^4^ cm^2^ mol^−1^). Due to the low temperature and positive ions, *F*_EA_ was increased from 23.4% to 35.6%, meaning the optimized conformation for the piezoelectricity.

As shown in Figure 3(b), the measured open-circuit voltage of the PENG ranged from 20 to 33 V in each compression. The device generated a positive and negative voltage during compression and when the structure was restored to its original position owing to the elasticity of the polyimide, respectively. As shown in the simplified schematic in Figure 3(c), the PENG in the TCO-HG is located between the top and bottom substrates. If the TCO-HG is operating on rigid ground, the PENG is expected to be compressed by the top substrate when an external vertical pressure is applied. In this case, the stress concentration on the hinge and mechanical strain caused by the vertical pressure induce an electrical potential difference between the two electrodes of the PENG, thereby generating an electrical output.

As mentioned in Figure 1(c), the proposed TCO-HG can rotate during vertical compression. The rotational TENG fabricated on the top of TCO-HG can harvest electrical energy from this rotation. The working mechanism of rotational TENG fabricated in this study is schematically illustrated in Figure S6 [45–47]. As shown in the figure, the bottom electrode has positive charge due the negative surface charge of PTFE. When the bottom electrode rotates, the electrons flow from the top electrode to the bottom electrode due to the surface charge of PTFE. As the bottom electrode continues to rotate, the electrode forms the left side contacts with the PTFE layer inducing electrons to flow from the bottom electrode. Therefore, alternative current is generated through rotation motion.

The rotational TENG requires constant contact and separation during the rotation to realize efficient power generation. Therefore, the degree of rotation of the top substrate is critical. The rotation angle of the TCO-HG can be calculated geometrically. If the length of line *AB*, *α*, the height, *h*, and the number of angles of the regular polygon *n* are known, the rotation angle of a regular-polygon TC can be calculated. *A*′ is the orthogonal projection of point *A*.

The length of line *A*′*Bm* can be obtained as

(9) $$\begin{array}{c} m=\sqrt{a^{2}-h^{2}}. \end{array}$$

*θ* is the rotation angle of the regular-polygon TC.

Moreover, *θ*_o_ can be defined as

(10) $$\begin{array}{c} \theta_{\text{o}}=\frac{2\pi }{n}-\theta . \end{array}$$

*R* can be expressed as

(11) $$\begin{array}{c} R=\frac{a/2}{\sin\left(\pi /n\right)}. \end{array}$$

According to the expression of the circumferential angle, ∠*A*′*PB* is *θ*_o_/2 and ∠*BA*′*P* is 90°. Therefore,

(12) $$\begin{array}{c} \frac{m}{2R}=\sin\left(\frac{\theta_{\text{o}}}{2}\right)=\sin\left(\frac{\pi }{n}-\frac{\theta }{2}\right). \end{array}$$

Consequently,

(13) $$\begin{array}{c} \theta =\frac{2\pi }{n}-2\arcsin\left(\frac{m}{2R}\right)=\frac{2\pi }{n}-2\arcsin\left(\frac{\sqrt{a^{2}-h^{2}}}{a}\sin\left(\frac{\pi }{n}\right)\right). \end{array}$$

Equation (13) can be used to perform the calculation, with *a* = 5 cm, *n* = 5, and *h* = 3.75 cm. In this case, *θ* is 26.242°, and thus, the total rotation angle *θ* of the four-story TC is 104.968°.

**Figure 4:**
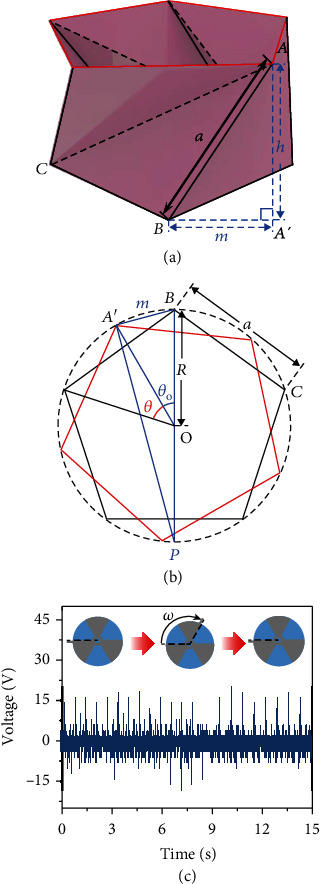
Schematic of single-stage TC and electrical performance of the rotational TENG. (a) Single-stage TC. (b) Base polygon of TC. (c) Open-circuit voltage of rotational TENG during operation.

The three electrodes on the top substrate were designed as 52° sectors to ensure the contact separation of the rotational TENG according to the calculated rotational angle. On the top electrode, a polytetrafluoroethylene (PTFE) film was applied to generate a surface charge during the contact with the bottom electrode. During the compression, the aligned top and bottom electrodes began separating horizontally as the bottom electrode rotated. When the TCO-HG was completely compressed, the top and bottom electrodes were completely separated. After the external pressure was removed, the bottom electrode rotated to its original position. Through continuous operation, the rotational TENG could produce an open-circuit voltage of 15 V (Figure 4(c)).

**Figure 5:**
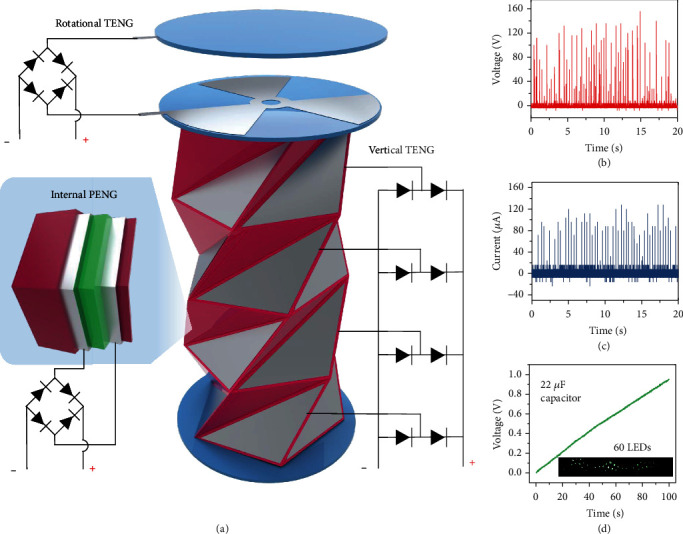
Schematic and electrical performance of the TCO-HG with a rectifier circuit. (a) Vertical TENG, rotational TENG, and PENG generator connected to the rectifier circuit. (b) Open-circuit voltage and (c) closed-circuit current output of the TCO-HG with the rectifier circuit. (d) TCO-HG charging a 22 *μ*F capacitor and powering 60 LEDs in a single compression.

As shown in Figure 5(a), the vertical TENG, internal PENG, and rotational TENG in the TCO-HG could be connected to a rectifier circuit to expand the device applicability. As shown in the photograph of the TCO-HG in Figure S7, each electrode was connected to a wire and rectifier circuit to convert the repeatedly applied vertical pressure into electricity. Each generator produced electrical output and could be combined with a rectifier circuit. As shown in Figures 5(b) and 5(c), the measured peak open-circuit voltage and closed-circuit current of the combined TCO-HG were 120 V and 90 *μ*A, respectively. For measuring mechanical stability of the device, TCO-HG was given input by 6 Hz for 48 hours. TCO-HG has shown consistent output over 48 hours of operation as shown in Figure S8. The TCO-HG could charge a 22 *μ*F commercial capacitor and power 60 LEDs (Figure 5(d)). In addition, total energy *W* and total charge *Q* stored in a capacitor can be described as

(14) $$\begin{array}{c} W=\frac{1}{2}CV^{2}=\frac{1}{2}VQ, \end{array}$$

where *C* is the capacitance and *V* is the voltage across the capacitor. By this equation, this device can generate 9.82 *μ*J of energy and 20.8 *μ*C of transferred charge for 100 seconds. The electrical output of TCO-HG can be further increased through stacking more stages of hybrid generator which would increase the electrical output generated by PENG/TENG hybrid generator and increase the rotation angle of rotational TENG.

## 3. Conclusions

We developed a TCO-HG that could harvest mechanical pressure. The device consisted of a vertical contact-separation TENG on the surface of a triangulate cylinder, PENG on the inner hinge, and rotational TENG on the top substrate to harvest mechanical energy from each motion. The base of the TC was fabricated as a pentagon to produce the maximum electrical output by maximizing the contact area of the TENG. On the inner hinge, on which a high material deformation could be induced, a PENG was fabricated to generate an electrical output. In addition, a four-stage TC was stacked to ensure a suitable rotating angle for harvesting rotational energy when the device was compressed. Through the power output of each generator, the device could successfully charge a 22 *μ*F commercial capacitor and power 60 LEDs each time the device was compressed. We believe that the proposed origami structure can be a potential design alternative for hybrid generators.

## 4. Materials and Methods

### 4.1. Materials

PVDF (molecular weight≃27.5 kg/mol), dimethyl sulfoxide (DMSO), and sodium chloride (NaCl) were obtained from Sigma-Aldrich (USA). All the reagents were used without further purification.

### 4.2. Piezoelectric PVDF Film Preparation

A piezoelectric PVDF film with sufficient *β*-phase content was synthesized based on the nonsolvent-induced phase separation process [43]. In this study, DMSO and NaCl were the good and nonsolvents of PVDF, respectively. A 30 wt% PVDF solution in the good solvent was prepared by heating at 100°C for 3 h. Subsequently, a doctor blade was adopted to render PVDF with a uniform thickness of 250 *μ*m over the glass substrate. The glass substrate with the PVDF was directly immersed in a 300 g/L NaCl coagulation bath, which was placed in a freezer at -20°C. The quenching process was performed for 30 min to induce sufficient phase inversion. The prepared piezoelectric PVDF films were washed thoroughly with deionized water and dried overnight.

### 4.3. Fabrication of Substrate for TCO-HG

The TCO-HG was composed of the origami structure and three parts: vertical contact TENG, rotational TENG, and PENG. The origami structure was based on a polyimide film with a thickness of 125 *μ*m, cut into a 20 cm × 25 cm parallelogram with an angle of 72°. The film was folded into a four-story pentagonal TC pattern, and 20 cm edges were glued together to form the cylinder. In particular, c lines formed two 2 cm × 0.1 cm hinges at an interval of 1.5 cm. Finally, bare acrylic plates with a diameter and thickness of 10 cm and 2 mm, respectively, were attached on both the bases of the TC.

### 4.4. Fabrication of Vertical Contact TENG

An isosceles triangle with a base and height of 6.5 cm and 2.4 cm, respectively, was fabricated. Commercial polyimide and aluminum tapes were attached on each upward and downward triangle, respectively.

### 4.5. Fabrication of Inner PENG

Commercial aluminum tapes cut into rhombus shapes with 4 cm × 3 cm diagonals were attached on both sides of the piezoelectric PVDF film, which was cut into a rhombus shape with 5 cm × 3.6 cm diagonals. These tapes were placed along the longer diagonal and inside of the c line.

### 4.6. Fabrication of the Rotational TENG

Two commercial aluminum tapes were cut into disk-shaped components with three sectors each, as shown in Figure 1(a). One aluminum tape was attached on the bare acrylic plate of the top base. The other aluminum tape was attached on a bare acrylic plate, and the PTFE film was placed on the surface of the Al tape. Finally, the two sheets were placed together, with the two surfaces connected securely. Both centers of the disks, in coincidence with the spinning axis, were fixed with screws.

## Data Availability

All other data are available from the corresponding authors upon reasonable request.

## Supplementary Materials

Supplementary Materials Figure S1: schematic illustration of working mechanism of vertical contact-separation TENG. Figure S2: 2D schematic of regular polygon composed of isosceles triangles. Figure S3: photograph of four-sided TC after one cycle of pressure. Figure S4: photograph of polyimide film (a) without slit hinge and (b) with slit hinge. Figure S5: characterization of poly(vinylidene) difluoride (PVDF) film. Figure S6: schematic illustration of working mechanism of rotational TENG. Figure S7: photograph of 4-stage TCO-HG. Figure S8: photograph of 4-stage TCO-HG. Table S1: Hansen solubility parameters of the ternary phase.
