# Supplementary material for: Triangulated Cylinder Origami-Based Piezoelectric/Triboelectric Hybrid Generator to Harvest Coupled Axial and Rotational Motion
Source: Research (Wash D C). 2021 Feb 19;2021:7248579. doi: 10.34133/2021/7248579 (PMC7914395; doi:10.34133/2021/7248579)
Supplement: Supplementary Materials — Figure S1: schematic illustration of working mechanism of vertical contact-separation TENG. Figure S2: 2D schematic of regular polygon composed of isosceles triangles. Figure S3: photograph of four-sided TC after one cycle of pressure. Figure S4: photograph of polyimide film (a) without slit hinge and (b) with slit hinge. Figure S5: characterization of poly(vinylidene) difluoride (PVDF) film. Figure S6: schematic illustration of working mechanism of rotational TENG. Figure S7: photograph of 4-stage TCO-HG. Figure S8: photograph of 4-stage TCO-HG. Table S1: Hansen solubility parameters of the ternary phase. [file 7248579.f1.docx]

**
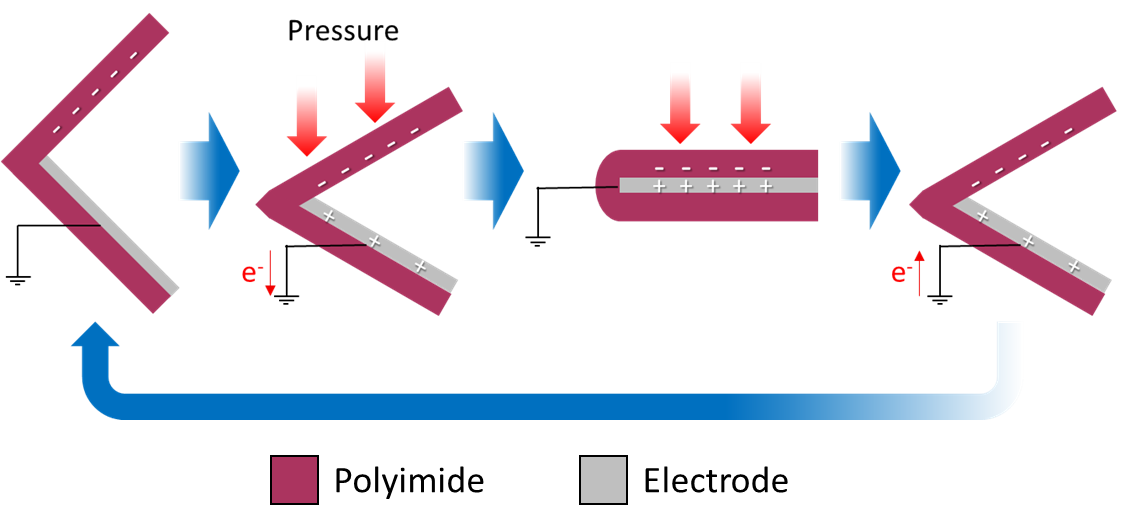
**

**Figure S1. Schematic illustration of working mechanism of vertical-contact separation TENG.**

**
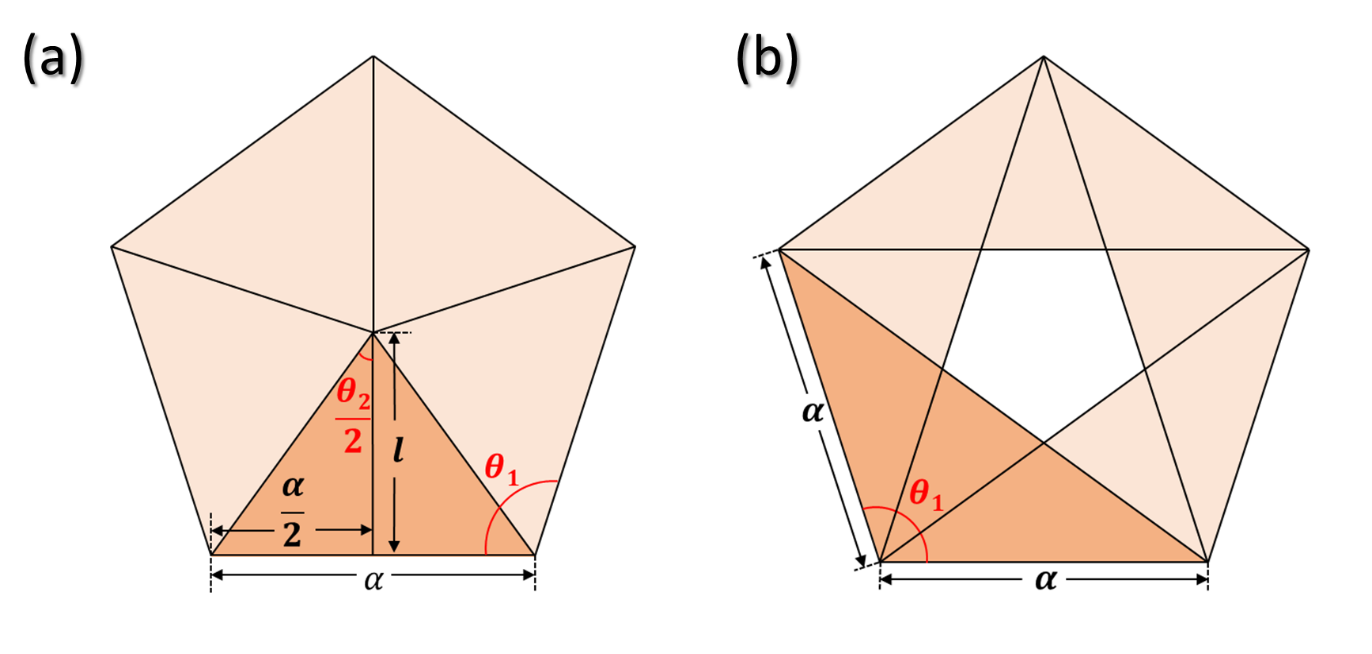
Figure S2. 2-D schematic of regular polygon composed of isosceles triangles.**


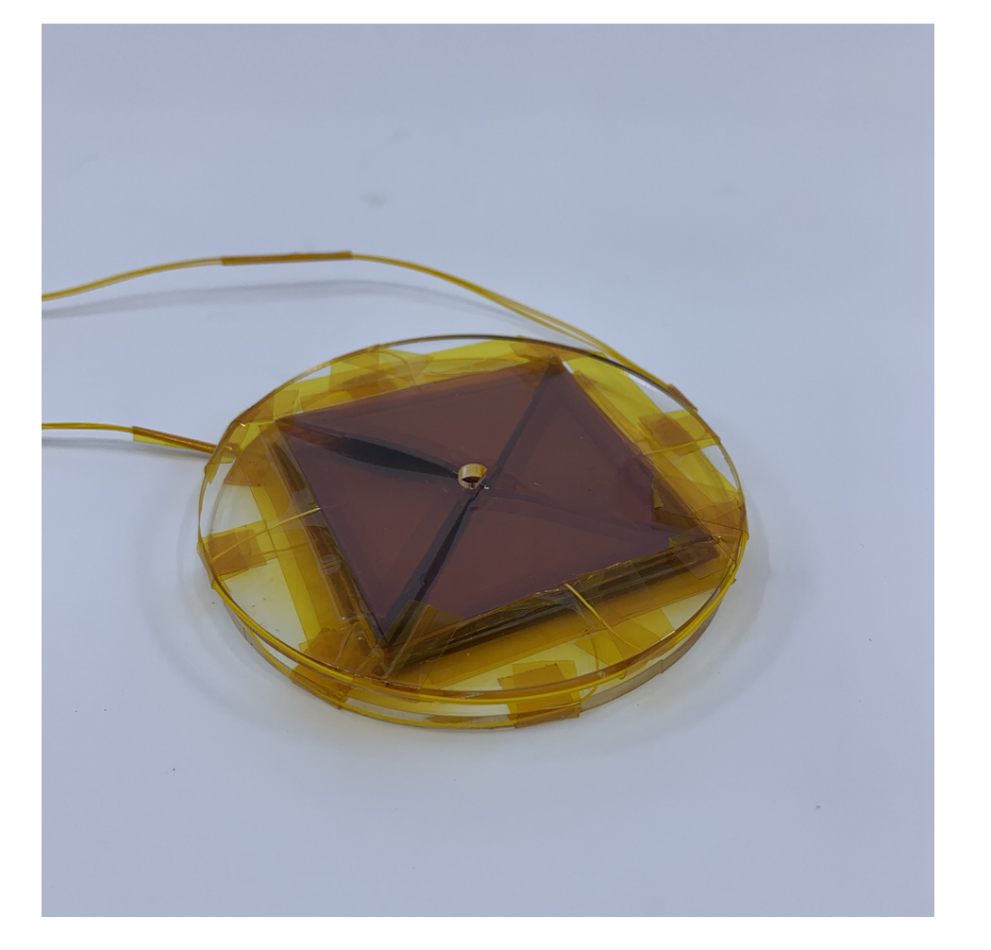


**Figure S3. Photograph of four-sided TC after one cycle of pressure.**

**
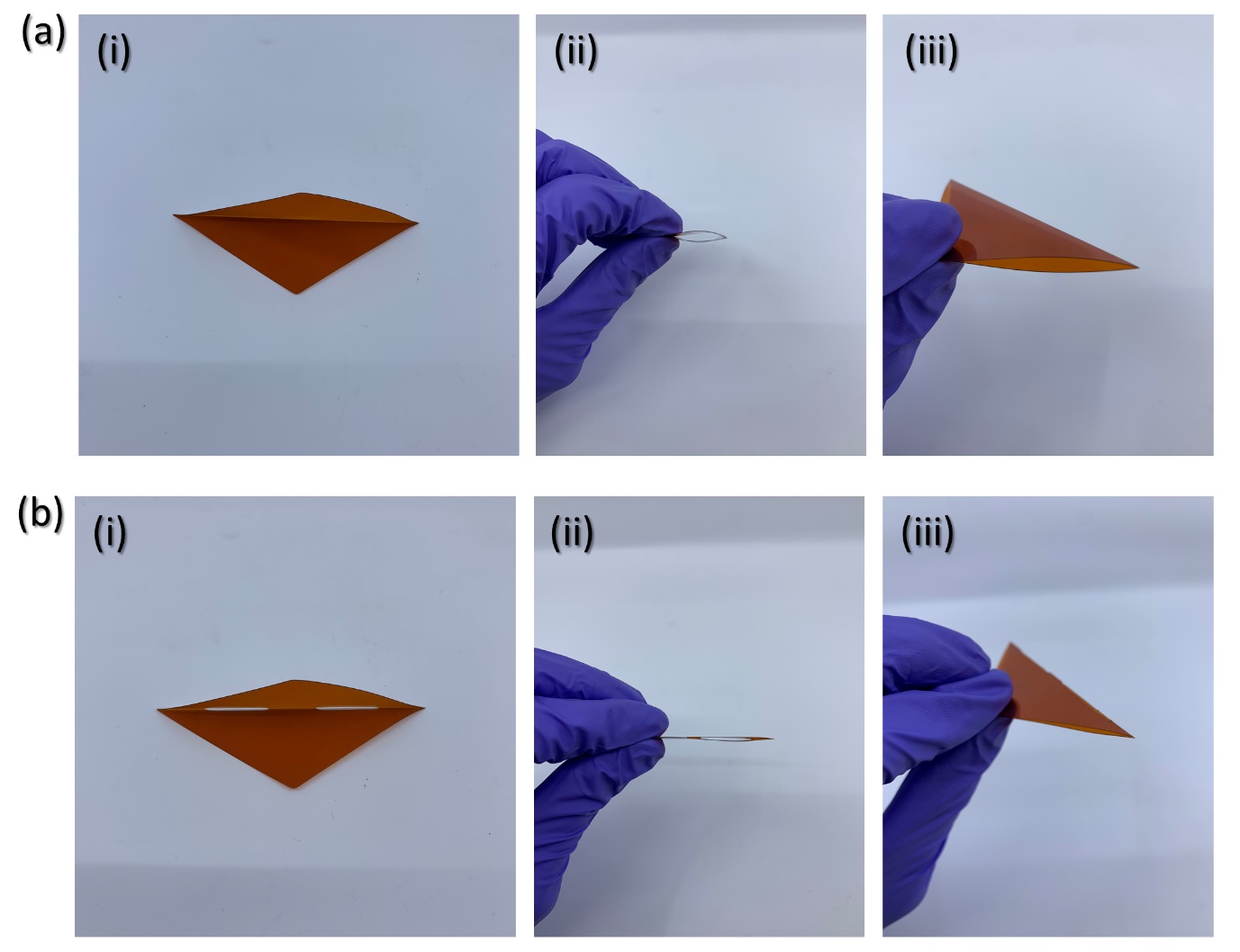
**

**Figure S4. Photograph of polyimide film (a) without slit hinge and (b) with slit hinge.**

**
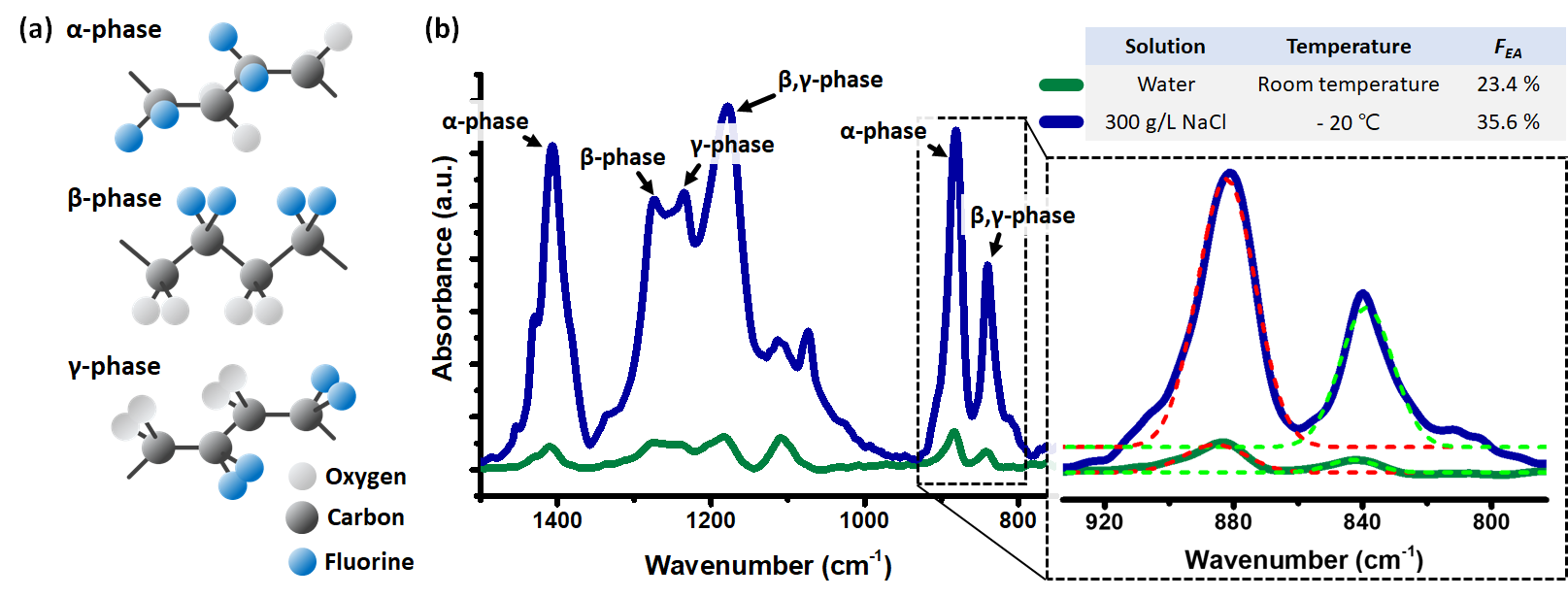
Figure S5. Characterization of poly(vinylidene) difluoride (PVDF) film.** (a) Chemical structures of α-phase, β-phase, and γ-phase of PVDF. (b) FT-IR spectrums of PVDF films fabricated within two coagulation baths, i.e., (green) general water coagulation bath, (blue) low-temperature ionic coagulation bath.


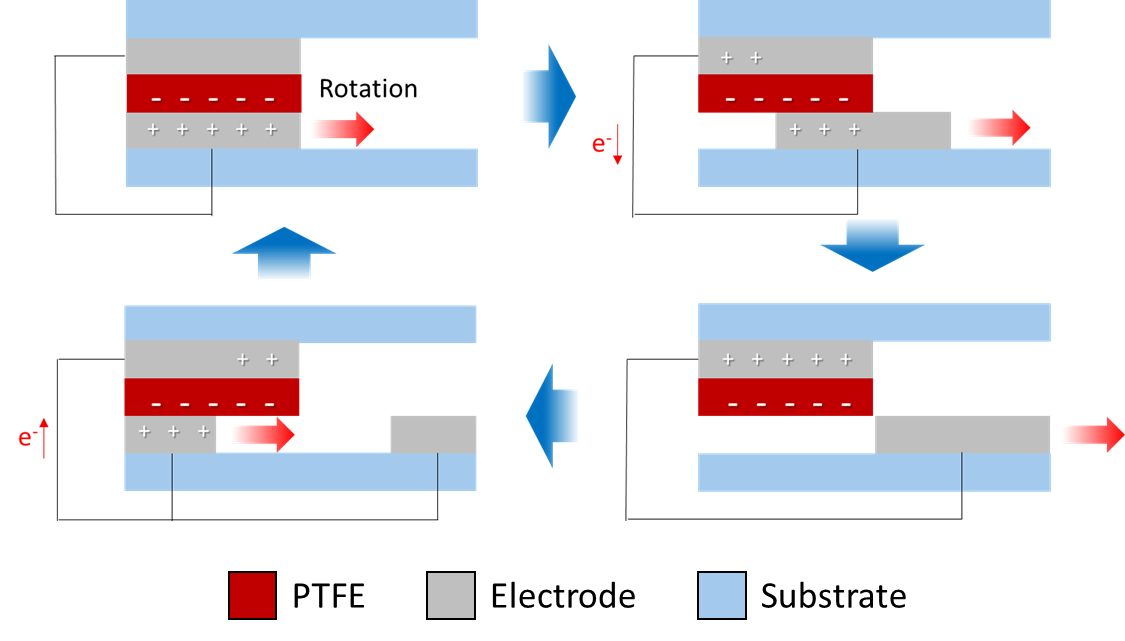


**Figure S6.** Schematic illustration of working mechanism of rotational TENG.

**
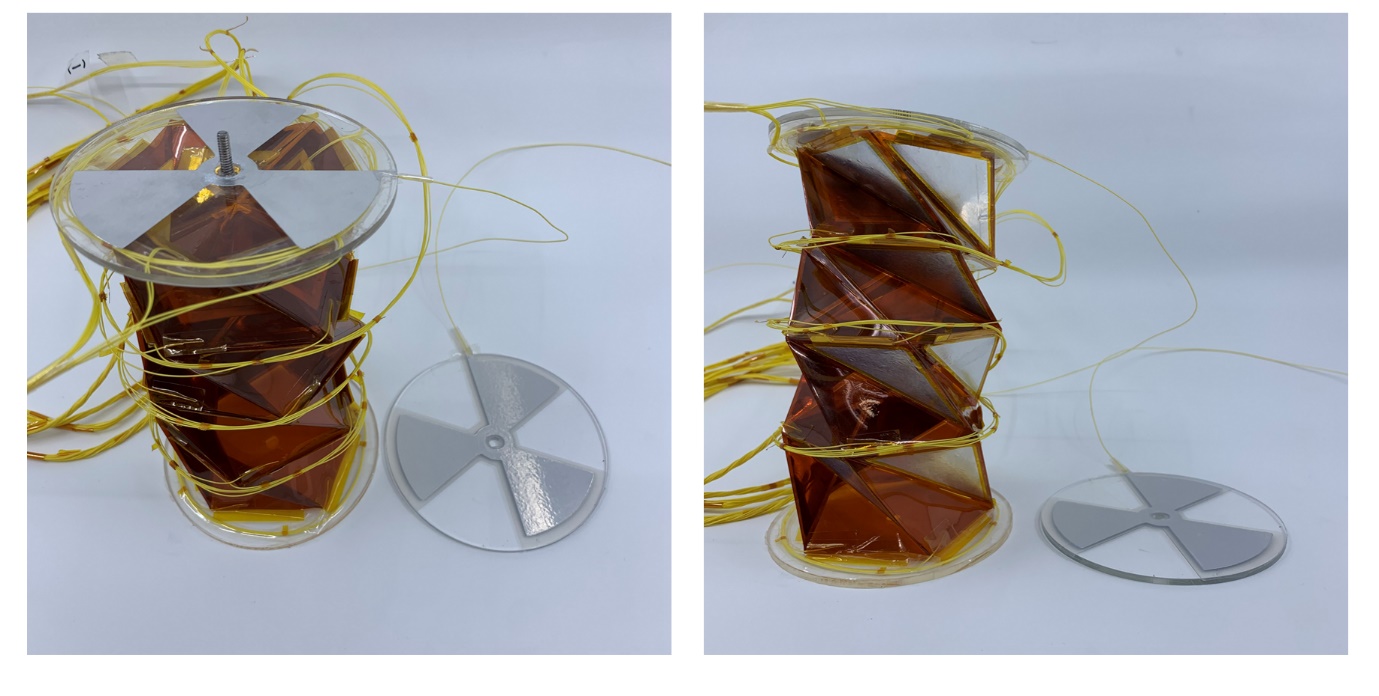
**

**Figure S7. Photograph of 4 stage TCO-HG**


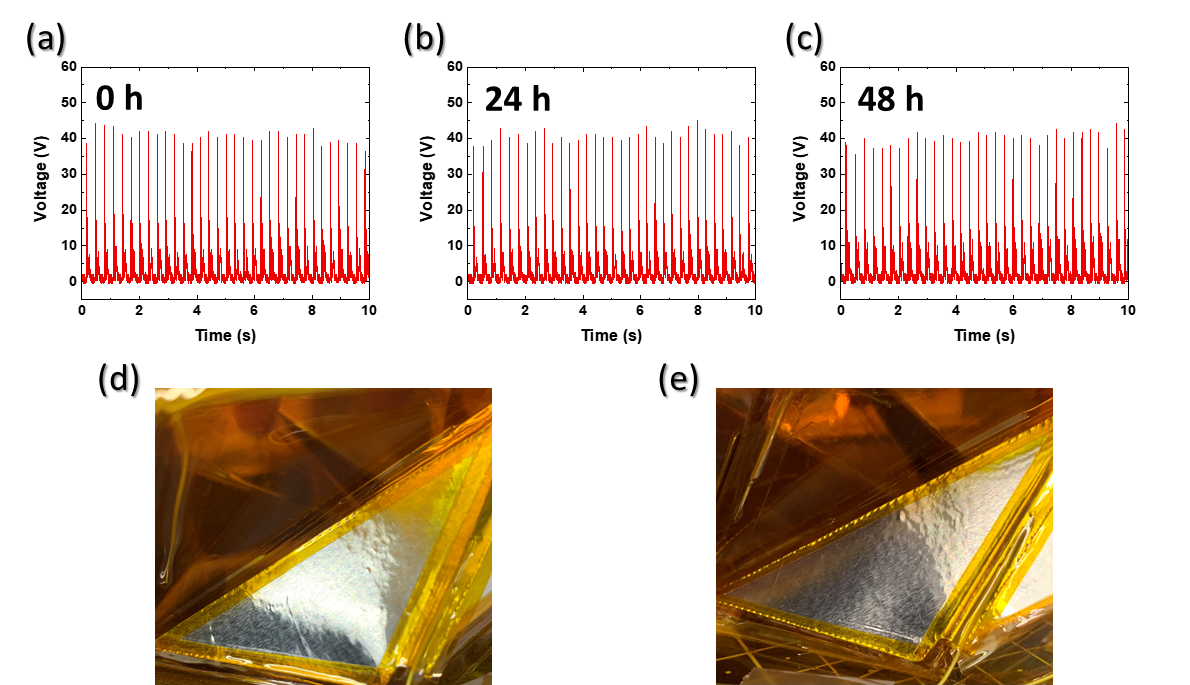


**Figure S8. Photograph of 4 stage TCO-HG**

Table S1. Hansen solubility parameters of the ternary phase; PVDF, DMSO, and water

|  | **Hansen solubility parameters [MPa^0.5^]** | | | | *** R_o_** | **** R_a_** | ***** RED** |
| --- | --- | --- | --- | --- | --- | --- | --- |
|  | **δ_d_** | **δ_p_** | **δ_h_** | **δ_total_** |  |  |  |
| **PVDF** | 17.2 | 12.5 | 9.2 | 23.2 | 10.5 | - | - |
| **DMSO** | 18.4 | 16.4 | 10.2 | 26.7 | - | 4.69 | 0.45 |
| **Water** | 15.6 | 16 | 42.3 | 47.8 | - | 33.4 | 3.18 |

* $R_{0}:Interaction radius of Hansen sphere$

** $R_{a}=\sqrt{4{(\delta_{d2}-\delta_{d1})}^{2}+{(\delta_{p2}-\delta_{p1})}^{2}+{(\delta_{h2}-\delta_{h1})}^{2}} (1:solvent, 2, polymer)$

*** $\mathrm{RED}\left( relative energy difference \right)=\frac{R_{a}}{R_{0}}$
